# Bubbles in my heart-systemic air embolism after CT-guided transthoracic biopsy of a pulmonary nodule

**DOI:** 10.36416/1806-3756/e20240227

**Published:** 2024-11-16

**Authors:** Felipe Marques da Costa, Augusto Kreling Medeiros, Felipe Roth Vargas

**Affiliations:** 1. Serviço de Pneumologia, Hospital Beneficência Portuguesa de São Paulo, São Paulo (SP) Brasil.; 2. BP Medicina Diagnóstica, Hospital Beneficência Portuguesa de São Paulo, São Paulo (SP) Brasil.; 3. Serviço de Radiologia Intervencionista, Hospital Beneficência Portuguesa de São Paulo, São Paulo (SP) Brasil.

**Figure 1:**
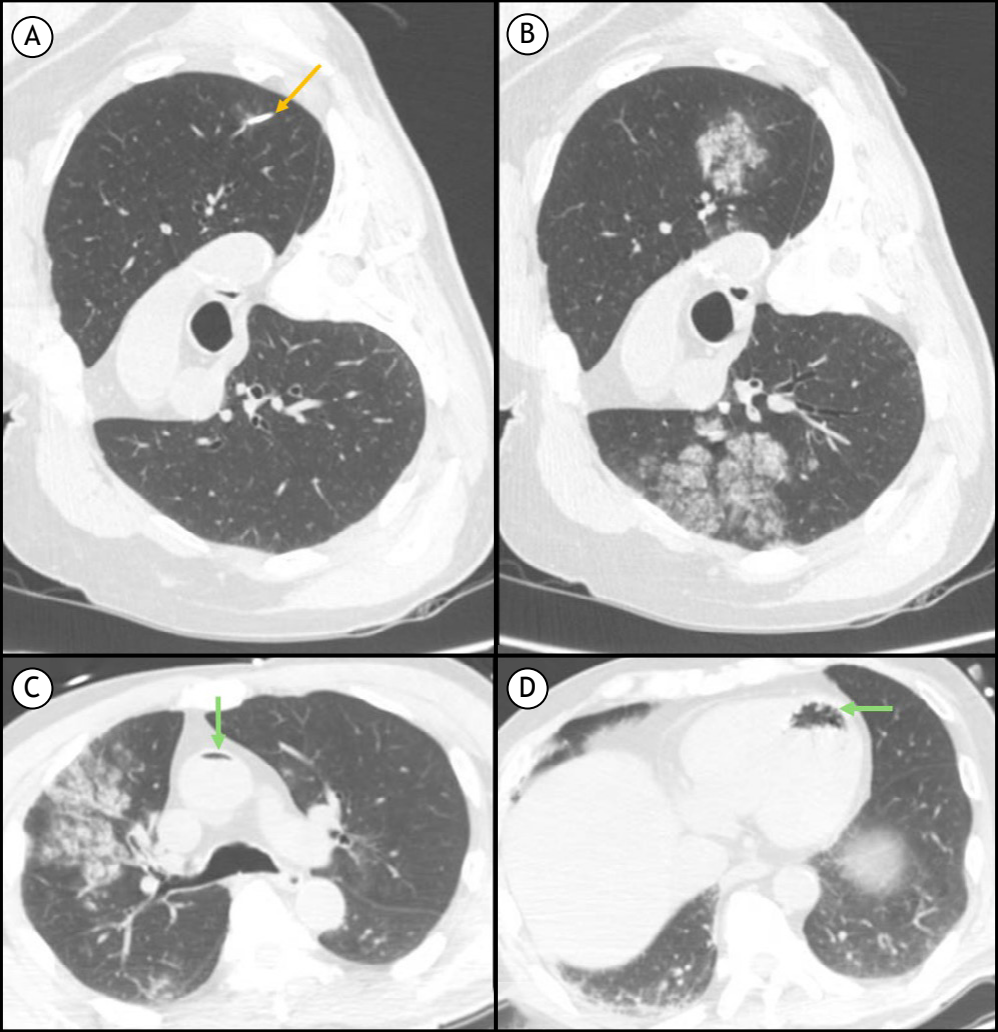
Axial CT slices. Slices A and B were acquired in the right lateral position. (A) The needle tip is within the lung nodule (orange arrow). (B) A few minutes after the needle biopsy, hemorrhage was observed surrounding the nodule in the left lung, as well as in the right lung, presumably due to contralateral aspiration favored by the right lateral position. Slices C and D were acquired in the supine position, at 15 m after the biopsy, and show a small amount of air in the ascending aorta, as well as air within the left ventricle (green arrows).

A 70-year-old male former smoker with a history of hypertension was admitted for elective CT-guided transthoracic biopsy (CTTB) of a suspicious pulmonary nodule. He was asymptomatic, and the physical examination was unremarkable. Unenhanced CT images from the periprocedural period are shown in Figure 1. The patient was diagnosed with a systemic air embolism (SAE), after which he was placed in the Trendelenburg position and received oxygen therapy for 3 days, being discharged without complications (Figure 1).

Although it is potentially fatal complication, CTTB-related SAE is rare, occurring in less than 0.1% of cases.^1^ ^-^ ^3^ The pathophysiology involves two primary mechanisms^3^: the entry of air into the pulmonary vein through the biopsy needle when atmospheric pressure exceeds pulmonary venous pressure; and the formation of a bronchovenous fistula. These events can lead to catastrophic outcomes such as myocardial infarction and stroke.^1^

Risk factors for SAE include a lesion being located in a lower lung lobe, a pulmonary nodule with a subsolid composition, pneumothorax, hemorrhage, and a lesion being located above the level of the left atrium.^2^ Preventive measures, such as placing the patient in a biopsy side-down position, have significantly reduced the incidence of this complication.^1^ Immediate postprocedural management involves administering oxygen to achieve an SpO_2_ near 100%, placing the patient in the Trendelenburg position, and applying hyperbaric oxygen therapy if available.^3^
